# Complete genome sequences of three *Porphyromonas gingivalis* strains, isolated from patient with esophageal squamous cell carcinoma and healthy individual in China

**DOI:** 10.1128/mra.00832-23

**Published:** 2024-05-08

**Authors:** Bianli Gu, Lixia Ma, Pan Chen, Yijun Qi, Qi Zhao, Shegan Gao

**Affiliations:** 1 Henan Key Laboratory of Microbiome and Esophageal Cancer Prevention and Treatment, Henan Key Laboratory of Cancer Epigenetics, Cancer Hospital, The First Affiliated Hospital (College of Clinical Medicine) of Henan University of Science and Technology, Luoyang, Henan, China; 2 State Key Laboratory of Oncology in South China, Collaborative Innovation Center for Cancer Medicine, Sun Yat-Sen University Cancer Center, Guangzhou, China; University of Maryland School of Medicine, Baltimore, Maryland, USA

**Keywords:** genomes, *Porphyromonas gingivalis*

## Abstract

Here, we report the complete genome sequences of three *Porphyromonas gingivalis*, one from patient with esophageal cancer (LyEC01), and the other two from periodontally healthy individuals (LyG-1 and LyG-2) in 2021 and 2023. The genome sizes of LyEC01, LyG-1, and LyG-2 were 2,408,275, 2,411,440, and 2,411,481 bp, respectively.

## ANNOUNCEMENT

*Porphyromonas gingivalis* is a key bacterium in the development of periodontitis (1). This Gram-negative, anaerobic bacterium has been implicated in the progression of various systemic diseases, including esophageal cancer (EC) (2, 3), and wild-type strains isolated from EC tissue have not been previously documented. For this study, plaque samples from a healthy volunteer and fresh tumor tissue from a patient with EC were collected from Central China in 2020. The samples were homogenized, streaked on blood plates (5% defibrated sheep blood) supplemented with 5 µg/mL hemin-menadione, and cultured anaerobically at 37°C for 7–14 days. The black colonies were picked, streaked, and cultured again after being identified by qPCR (4). Repeat the process at least two rounds to obtain purified colonies.

Gram staining, qPCR, and 16S rRNA sequencing were performed on the cultures, followed by genomic DNA extraction using E.A.N.A. Bacterial DNA kit (Omega). The Illumina library preparation involved randomly fragmenting 200 ng of the genomic DNA to <500 bp by sonication (Covaris S220), followed by using the VAHTS Universal DNA Library Prep Kit (Vazyme). Sequencing was carried out on the NovaSeq platform using a 2 × 150 bp paired-end configuration and a coverage of 100×. The data obtained were analyzed by the NovaSeq Control Software, Off-Line Basecaller (OLB), and GAPipeline-1.6 (Illumina). Quality filtering of the raw data were done using fastp v0.23.0 (5) to remove adapter, low-quality reads (Q20 <40%), poly-N (>14 bp), and error pair-end reads. For PacBio, 5–10 µg genomic DNA was sheared using a g-TUBE device (Covaris). The library was prepared using a SMRTbell prep kit 3.0 (PacBio), quantified and validated by Qubit 3.0 (Invitrogen) and 2100 Bioanalyzer System (Agilent Technologies) after being purified by AMPure PB Beads. The HiFi reads derived from PacBio system (6) were directly assembled using HGAP4/Falcon of WGS-Assembler 8.2 (7, 8) for LyG-1 and LyG-2, Hifiasm (v0.13-r308) (9), and Canu v1.7 (6) for LyEC01 without QC, error correction, and other processing. Assembly errors were corrected using Pilon v1.22 (https://github.com/broadinstitute/pilon). Default settings were used for all software, unless otherwise noted. Circular genomes were defined through blast alignments and the *dnaA* gene. Gene annotation was performed using the NCBI Prokaryotic Genome Automatic Annotation Pipeline (PGAP). Prophage sequences were predicted using the PHASTER web server (10).

The genomes of LyEC01, LyG-1, and LyG-2 each consist of a single chromosome (2.4 Mb) and one prophage. Table 1 provides detailed information.

**TABLE 1:** Genomic characteristics of three *P. gingivalis* strains isolated from an esophageal cancer patient and a dental healthy volunteer

Strains	Genome size (bp)	No. of Novaseq clean reads	Mean depth (×)	No. of PacBio clean reads	Long-read N50 (bp)	Genome assembly N50 (bp)	GC content (%)	Total genes	Regions of CRISPR	Total prophage length (bp)	Genome assembly accession no.
LyEC01	2,408,275	9,593,952	71.46	29,677	7,391	2,408,275	48.3	2,068	4	10,000	GCA_030144345.1
LyG-1	2,411,440	11,599,888	325.95	158,345	6,580	2,411,440	48.3	2,105	3	10,900	GCA_018141765.1
LyG-2	2,411,481	9,484,114	276.08	134,682	6,612	2,411,481	48.3	2,103	3	10,900	GCA_018141745.1

## Data Availability

The 16S rRNA sequences have been submitted to GenBank under accession numbers OR206419 (LyEC01), OR206492 (LyG-1), and OR206493 (LyG-2). The assembled genomic sequences can be found in GenBank under the accession numbers CP126309 (LyEC01), CP073349 (LyG-1), and CP073350 (LyG-2).The BioProject accession numbers are PRJNA974917 (LyEC01), and PRJNA722847 (LyG-1 and LyG-2). Raw data for Illumina are available with SRA accession numbers SRR25014312 (LyEC01), SRR24973794 (LyG-1), and SRR24973795 (LyG-2), while PacBio reads can be accessed with numbers SRR25015396 (LyEC01), SRR25017226 (LyG-1), and SRR25017225 (LyG-2).
